# Development of a multi-source blue light irradiation platform for photometric quantification of ascorbic acid *via* continuous absorbance quenching of the ferric–thiocyanate complex

**DOI:** 10.1039/d5ra07160g

**Published:** 2025-10-24

**Authors:** Ghufran K. Allawi, Turkey N. S.

**Affiliations:** a Department of Chemistry, College of Science, University of Baghdad Baghdad Iraq nagham.turkey@sc.uobaghdad.edu.iq; b College of Food Science, Al-Qasim Green University Babylon Iraq ghufran.k.allawi@fosci.uoqasim.edu.iq

## Abstract

A novel and meticulously optimised continuous flow injection analysis (CFIA) protocol was developed for the spectrophotometric quantification of ascorbic acid using a locally engineered optical system. The detection unit incorporated eight blue light-emitting diodes (LEDs) arranged in a matrix geometry distributed across two angular regions: 0–90° and 0–180°, while dual solar cells served as photonic detectors. Spectral measurements were specifically conducted at 0–180°, yielding enhanced absorbance stability and signal quality. Based on a redox-induced quenching mechanism, the system utilises a chromogenic FeSCN^2+^ complex, formed *in situ* through the controlled mixing of ferric (Fe^3+^) and thiocyanate (SCN^−^) ions under strictly maintained physical and chemical conditions. Upon interaction at the Y-point junction, injected ascorbic acid reduces the complex, yielding a quantifiable decrease in absorbance intensity proportional to its concentration. A linear calibration range was established from 0.5 to 40 mmol L^−1^ (*r*^2^ = 0.9986), LOD of 0.05 mmol L^−1^ (1.3209 μg per sample) (S/N = 3) and LOQ of 0.131 mmol L^−1^ (S/N = 10). Method precision was evaluated at two concentrations, 12 and 30 mmol L^−1^, showing intra-day RSD% < 1% and inter-day RSD% < 2%, affirming excellent repeatability and method reliability. Validation was extended to three commercial pharmaceutical preparations containing 500 mg of ascorbic acid using the standard addition method. Comparative assessment against UV spectrophotometry and HPLC, with statistical evaluation *via* Student's *t*-test and one-way ANOVA, confirming no significant differences among techniques (*t*_cal < *t*_tab; *F*_cal < *F*_tab, *α* = 0.05), with recovery values between 97.13% and 103.6%.

## Abstract 2

Diagram of CFIA system: (A) Fe^3+^ line; (B) SCN^−^ line; (C) A.A carrier; (D) sample valve; (E) mixing/reaction coil quenched by A.A; (F) blue LED detector.

## Graphical abstract

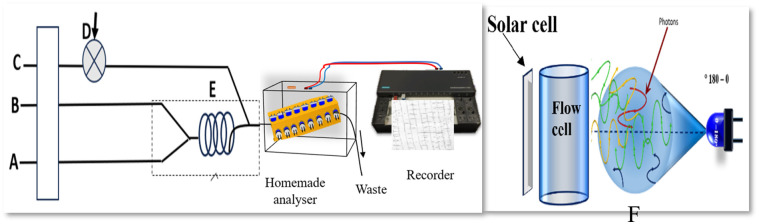

## Introduction

Ascorbic acid (AA), widely recognised as vitamin C, is an organic compound that is soluble in water, and characterised by the chemical formula C_6_H_8_O_6_. It contains a lactone ring and an enediol structure, which confer substantial reducing and antioxidant properties. AA is moderately acidic (p*K*_a_ ≈ 4.2), highly soluble in aqueous media, and undergoes reversible oxidation to dehydroascorbic acid (DHAA), followed by irreversible degradation under oxidative conditions. These redox characteristics are central to its biological and analytical behaviour. In biological systems, AA plays essential roles in collagen biosynthesis, iron absorption, immune modulation, and neurotransmitter production. It functions as a cofactor in hydroxylation reactions and contributes to cellular protection against oxidative stress. Deficiency in AA leads to scurvy, which is characterised by impaired connective tissue integrity, gingival bleeding, and vascular fragility. Excessive intake, particularly through supplementation, may result in gastrointestinal discomfort, oxalate nephrolithiasis, and conditional pro-oxidant effects under specific metabolic conditions.^1,2^ Due to its physiological importance and widespread use in pharmaceutical formulations, accurate quantification of AA is critical in clinical, nutritional, and industrial contexts. Various analytical techniques have been developed for this purpose, including spectrophotometry,^3^ high-performance liquid chromatography (HPLC),^4^ electrochemical sensing,^5^ chemiluminescence,^6^ and enzymatic assays.^7^ These methods often exploit AA's reducing capacity or its ability to form complexes with metal ions or chromogenic reagents. Several spectrophotometric and flow-based methods have demonstrated high sensitivity and selectivity. For example, a green flow injection spectrophotometric method utilised a natural reagent extracted from *Areca catechu*, where AA reduced Fe(iii) to Fe(ii), and the remaining Fe(iii) formed a colored complex with the reagent at 560 nm^3^. Another study employed Cu(ii)–neocuproine as an oxidising system, where AA was oxidised to DHAA, and the absorbance of the resulting complex was measured at 450 nm.^8^ A spectrofluorimetric method based on the reduction of Fe(iii) followed by complexation with 2,4,6-tripyridyl-*s*-triazine (TPTZ) yielded a fluorescent Fe(ii)–TPTZ complex measurable at 790 nm.^9^ Additionally, a method using potassium dichromate and permanganate as oxidants demonstrated strong absorbance shifts upon AA reduction, with detection at 350 nm and 530 nm, respectively.^10^ Flow injection analysis (FIA) has proven to be a highly effective technique for determining pharmaceutical compounds and ionic species.^11–19^ It offers rapid analysis, low reagent consumption, compatibility with automation, and reliable reproducibility. FIA systems can be integrated with custom-built mixing coils and diverse detection modes, making them particularly suitable for high-throughput pharmaceutical applications and complex sample matrices.^20–23^ To address the analytical challenges associated with precise and efficient AA quantification, this study introduces a locally engineered spectrophotometric flow system based on structured optical irradiation. The system uses eight adjacent blue light sources (1.5 W each), arranged as a visible matrix, to irradiate a continuous flow tube (outer diameter, 4 mm; inner diameter, 2 mm). Detection is achieved using twin solar cells (each 37.8 mm in size) positioned at 0° and 180° relative to the irradiation zone. The transmitted light intensity is enhanced by an internal optical fibre phenomenon occurring within the flow tube. Representative response profiles were recorded using a graphical plotter for the FeSCN^2+^ complex formation and its quenching by AA.

The novelty of the present approach lies in the development of a low-cost, multi-source blue light irradiation FIA platform integrated with dual solar cell photodetectors for continuous absorbance quenching monitoring of the Fe(iii)–thiocyanate–ascorbic acid system. Unlike the previously reported microfabricated glass-chip FIA system based on thermal-lens microscopy by Kitamori *et al.*^24^ the proposed method eliminates the need for complex laser alignment and costly instrumentation. The use of multidirectional blue LED illumination enhances optical interaction within a simple brass-housed flow cell, achieving comparable sensitivity (LOD 0.05 mmol L^−1^) while consuming only 150 μL of sample. This environmentally friendly and reproducible design represents a sustainable and accessible alternative consistent with green analytical chemistry principles.

## Experimental

### Reagents & chemicals

All reagents used in this study were of analytical grade and prepared using deionised water. A 0.050 mol per L solution of ascorbic acid (C_6_H_8_O_6_, MW = 176.12 g mol^−1^) was prepared by accurately weighing 2.202 g and dissolving it in deionised water to a final volume of 250 mL. The compound was obtained from BDH Chemicals Ltd. (Poole, England) and stored in an amber bottle to prevent photodegradation. A 0.100 mol per L solution of ferric chloride hexahydrate (FeCl_3_·6H_2_O, MW = 270.30 g mol^−1^) was prepared by dissolving 13.515 g in deionised water and diluting to 500 mL. Potassium thiocyanate (KSCN, MW = 97.18 g mol^−1^) was prepared at the same concentration (0.100 mol L^−1^) by dissolving 4.859 g in deionised water and completing the volume to 500 mL. BDH Chemicals Ltd supplied both reagents. Hydrochloric acid solution (1000 mol L^−1^) was prepared by diluting concentrated HCl obtained from BDH, which has a density of 1.18 g mL^−1^ and contains 36% w/w HCl. To prepare 500 mL of 1.000 mol per L HCl, 42.9 mL of the concentrated acid was carefully diluted with deionised water. The resulting solution was standardised by titration against a primary standard solution of sodium carbonate (Na_2_CO_3_, MW = 105.99 g mol^−1^) to ensure accuracy. All solutions were freshly prepared and stored in tightly sealed containers at room temperature. Light-sensitive reagents, particularly ascorbic acid, were protected from light exposure to maintain stability throughout the experimental procedures.

### Apparatus

**Fig. 1:**
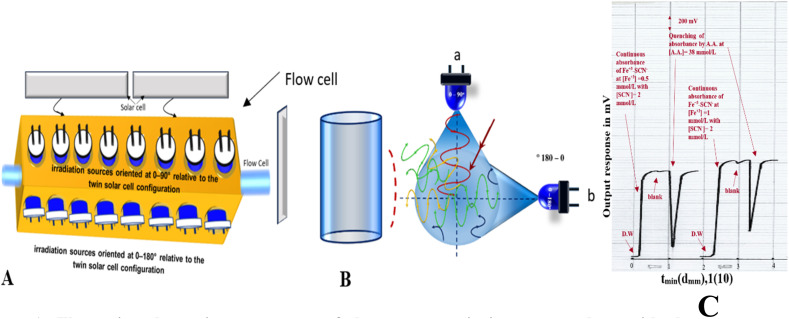
Illustrating the main components of the spectroscopic instrument along with the measurement configuration: (A) measurement cell, (B) illuminated area by: (a) source at 0–90°, (b) source at 0–180, (C) real profile measured responses obtained from the chart recorder (*T*: time in minute, *d*: distance in millimeter).

A homemade photometric instrument was employed as the primary photometric detection system.^25^ The device consists of a brass incubator with dimensions of 100 mm (length) × 40 mm (width), featuring eight perforations on one side that extend to contact the tubular flow cell. These perforations are positioned at an angle of 0° to 180° and are aligned with a twin solar cell unit used for signal detection. An additional set of eight perforations, identical in size, is located at an angle of 0° to 90° to accommodate blue light-emitting diode (LED) irradiation sources. The system allows flexible operation: either the 0–90° or 0–180° irradiation site may be used independently, or both may be combined to exploit spectral symmetry interference or self-absorption interference principles. Fig. 1 illustrates the structural details of the photometric instrument.

The locally engineered optical unit, termed in this work as the Dual-Angle Photonic Irradiation Spectrometer (DAPI-Spec) (Fig. 1A and B), is designed to operate on the principle of angular photonic excitation using high-intensity visible light sources. The system incorporates two independent LED arrays, each consisting of eight blue-emitting diodes (1.5 W, 5 mm diameter), embedded at a depth of 14 mm within a precision-machined brass block selected for its mechanical stability and ease of fabrication. The first array is positioned at an angular orientation of 0–90°, while the second is aligned at 0–180°, both relative to the detection axis.

Detection is achieved *via* dual solar cells, each measuring 37.8 mm, strategically placed to capture photonic output and compensate for attenuation and angular deviation effects caused by the sample matrix. The interaction between the irradiated light and the analyte flowing through the transverse flow cell generates a measurable signal, which is transmitted through the detector interface.

Each LED array operates independently and supports four adjustable irradiation levels, controlled *via* voltage modulation. This allows precise tuning of the incident photon flux (*P*^0^), defined as the number of photons striking a unit area per unit time. The intensity can be configured uniformly or variably across the array, depending on the nature of the analyte and the expected reaction dynamics. This modular control offers a distinct advantage over conventional commercial spectrophotometers, which typically lack such flexibility in excitation geometry and intensity modulation.

The system's architecture enables selective or combined use of the 0–90° and 0–180° configurations, facilitating advanced optical interference techniques such as spectral symmetry modulation and self-absorption correction. These features enhance signal resolution and analytical depth, particularly in complex matrices. Detailed schematics and representative signal profiles are presented in Fig. 1C, along with expanded interpretations of the acquired data, illustrating the system's capacity for high-fidelity spectrophotometric analysis across diverse applications.

The flow injection system included a four-channel peristaltic pump (IPC-N) that operated at adjustable flow rates, along with a six-port medium-pressure injection valve (7725i) featuring a sample loop constructed from Teflon tubing (1 mm i.d., variable length). Signal acquisition employed a potentiometric recorder (REC-10; 1–5 V range) for displaying output profiles and model files. For comparative analysis, use High-Performance Liquid Chromatography (HPLC) requires a binary solvent delivery system (LC-20AD), manual or automated injector (7725), analytical column (VP-ODS, 250 mm × 4.6 mm, 5 μm), UV detector (SPD-20A), and data acquisition software (LabSolutions). These components are essential for obtaining accurate separation, quantification, and spectral validation of analytes under standard chromatographic conditions.

### Methodology

**Fig. 2:**
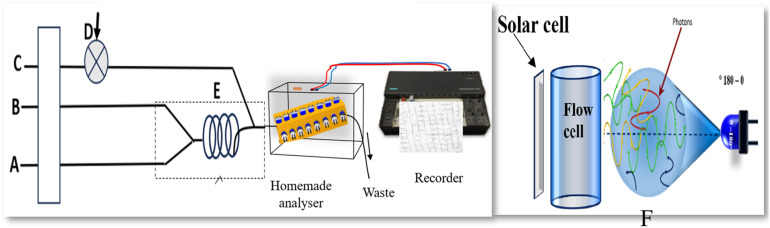
Diagram of the continuous flow injection system. (A) line moving ferric ion in hydrochloric acid medium; (B) line carrying thiocyanate ion; (C) carrier stream containing ascorbic acid; (D) injection valve for sample introduction; (E) the unit mixes reagents to create the red FeSCN^2+^ complex. This stage is followed by a reaction coil for complex development, which intersects line C to quench the complex with ascorbic acid prior to photometric detection. (F) The LED-blue irradiation source is shown with the single solar cell detector, which is positioned at a measuring angle of 0° to 180°.

The (CFIA) system (Fig. 2) was employed for the spectrophotometric determination of ascorbic acid. The flow manifold consisted of three channels: two allocated for reagent introduction and one designated as the carrier stream. The first reagent line delivered an aqueous ferric ion solution (2.0 mmol L^−1^), while the second conveyed thiocyanate ions (4.0 mmol L^−1^); both streams were propelled at synchronised flow rates of 1.6 mL min^−1^. These reagent solutions converged within a reaction coil (60 cm in length, 471 μL internal volume), facilitating the rapid and continuous *in situ* formation of the chromogenic FeSCN^2+^ complex. This deeply red complex produced a stable and reproducible absorbance signal throughout the detection path. The third channel introduced a 150 μL aliquot of ascorbic acid *via* a conventional injection valve, employing distilled water as the carrier at a flow rate of 1.5 mL min^−1^. Upon reaching the Y-shaped junction (Y-POINT), the ascorbic acid sample encountered the preformed FeSCN^2+^ complex, triggering a reductive interaction that disrupted its electronic structure and attenuated its absorbance intensity. Ascorbic acid concentration in the sample was directly correlated with the intensity of signal quenching. Deionised water served as an analytical blank to maintain baseline accuracy. Spectrophotometric measurements were carried out using a locally designed optical system that included eight blue light-emitting diodes (LEDs) as irradiation sources. Light energy was detected using a twin solar cell setup, which acted as a photonic energy converter. The system performed scans across a range of angles from 0° to 180°. Each sample was measured three times to ensure reproducibility and statistical reliability, and the absorbance responses were recorded using a calibrated strip-chart recorder.

The signal acquisition and processing workflow in the developed system proceeds as follows:

(1) Irradiation: the analyte flows through a 2 mm optical path within the transverse flow cell and is irradiated by blue LEDs (*λ* = 460 nm) positioned at 0–90° and/or 0–180°.

(2) Reaction principle: ascorbic acid reduces Fe^3+^ to Fe^2+^, suppressing the formation of the red [Fe(SCN)]^2+^ complex. This results in a decrease in absorbance intensity.

(3) Detection: the transmitted light is captured by dual solar cells, which convert photonic energy into analog electrical signals.

(4) Signal conversion: these signals are amplified and digitized *via* an analog-to-digital converter (ADC) integrated into a microcontroller.

(5) Quantification: the digital output (in millivolts) is directly proportional to the analyte concentration. A calibration curve using known standards is used to establish the concentration–response relationship.

## Results and discussion

### Experimental configuration for FeSCN^2+^ quenching-based spectrophotometric assessment of ascorbic acid

As explained before, the spectrophotometric detection of ascorbic acid in the developed CFIA system relies on two key chemical reactions. The first involves the coordination between ferric ions (Fe^3+^) and thiocyanate ions (SCN^−^), resulting in the formation of a highly coloured red complex,Fe^3+^ + SCN^−^ → FeSCN^2+^

This complex is characterised by its strong intramolecular charge-transfer absorption, which produces a continuous and stable absorbance signal under uninterrupted flow conditions. The intense red colouration reflects the electronic transitions within the FeSCN^2+^ species, making it an effective chromogenic probe. Upon injection of the ascorbic acid segment at the Y-shaped mixing zone, a reductive reaction occurs wherein the Fe^3+^ within the complex is reduced to Fe^2+^. The chromophoric structure is subsequently degraded, resulting in absorbance quenching proportional to analyte concentration. This process is governed by the following redox reaction:FeSCN^2+^ + C_6_H_8_O_6_ → Fe^2+^ + SCN^−^ + C_6_H_6_O_6_ + 2H^+^

The quenching effect arises from the disruption of the charge-transfer complex and the conversion of Fe^3+^ to Fe^2+^, which lacks the characteristic absorbance associated with FeSCN^2+^. Fig. 1C presents the recorded absorbance response, showing the stable and continuous signal produced by the FeSCN^2+^ complex in the absence of ascorbic acid and the pronounced decline upon sample injection. This decline is contrasted with the negligible effect caused by distilled water, which produces a minimal and statistically insignificant absorbance shift. These spectral patterns confirm the specificity and sensitivity of the system toward ascorbic acid detection through redox-induced absorbance modulation.

### Optimization of chemical and physical variable on absorbance profile and quenching response

#### Effect of ferric ion and thiocyanate ion concentrations

**Fig. 3:**
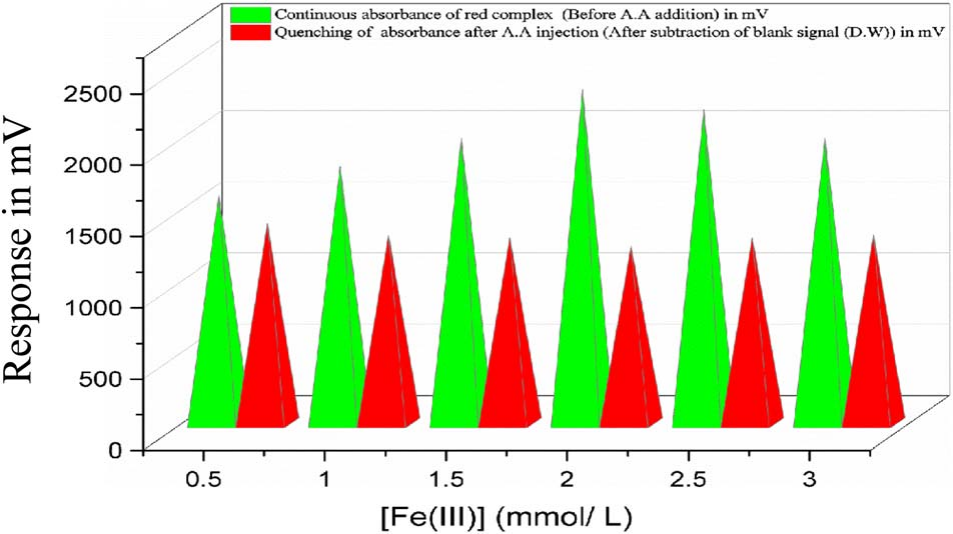
Effect of ferric ion concentration using Fe^3+^ (variable concentration)–SCN (2 mmol L^−1^)–H_2_O system and [A.A] = 38 mmol L^−1^ on absorbance of complex formation, quenching sensitivity by ascorbic acid and the optimal concentration of ferric ion = 2.0 mmol L^−1^.

The effect of varying ferric ion (Fe^3+^) concentration on the continuous absorbance of the FeSCN^2+^ complex was investigated using a flow injection analysis system. A series of Fe^3+^ solutions ranging from 0.5 to 3.0 mmol L^−1^ were prepared and introduced through the first channel of the system, while the thiocyanate ion (SCN^−^) concentration was held constant at 2 mmol L^−1^. Each solution was injected at a sample volume of 40 μL through the third stream, where ascorbic acid was delivered *via* distilled water as the carrier. The results revealed that the highest absorbance was achieved at an Fe^3+^ concentration of 2.0 mmol L^−1^ (Fig. 3). Beyond this concentration, a noticeable decline in absorbance was observed. This reduction is likely attributed to the formation of secondary complexes such as Fe(SCN)_2_^+^ and Fe(SCN)_3_, which exhibit lower molar absorptivity or are nearly colourless. Additionally, excess Fe^3+^ consumes available SCN^−^, shifting the equilibrium away from FeSCN^2+^ formation. These findings are consistent with previous spectrophotometric studies on the equilibrium behaviour of Fe^3+^–SCN^−^ systems.^26,27^ Therefore, an Fe^3+^ concentration of 2.0 mmol L^−1^ is recommended as the optimal condition for future analytical applications involving continuous absorbance monitoring of FeSCN^2+^.

**Fig. 4:**
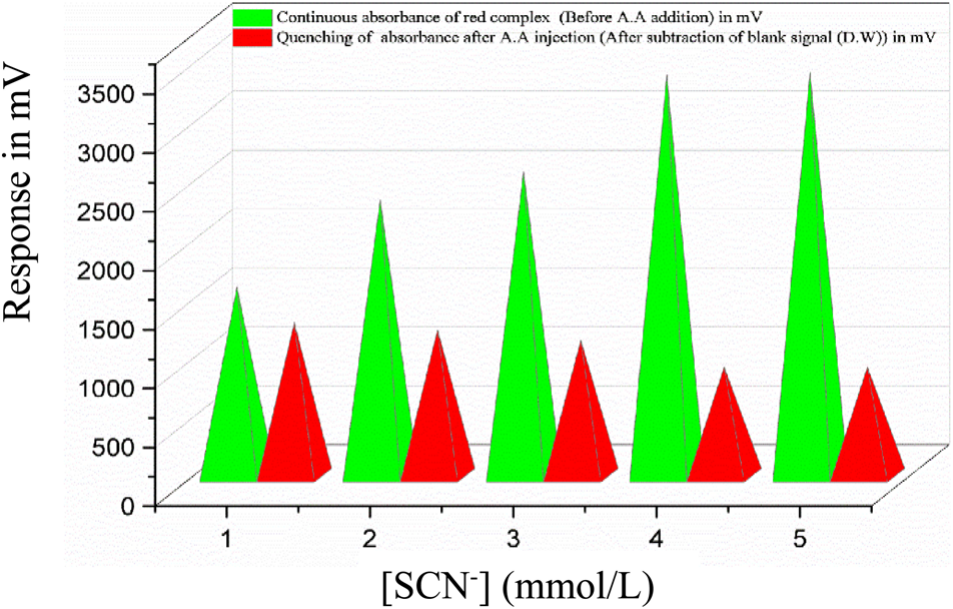
Effect of thiocyanate ion concentration using Fe^3+^ (2 mmol L^−1^)–SCN^−^ (variable concentration)–H_2_O system and [A.A] = 38 mmol L^−1^ on absorbance of complex formation, quenching by ascorbic acid and the optimal concentration of SCN^−^ = 4.0 mmol L^−1^.

A series of SCN^−^ solutions ranging from 1.0 to 5.0 mmol L^−1^ was prepared to examine its influence on the continuous absorbance response of the FeSCN^2+^ complex under flow injection conditions. The absorbance increased steadily with SCN^−^ concentration up to 4.0 mmol L^−1^, after which no significant enhancement was observed. This plateau suggests that the system may have reached a saturation point, where further addition of SCN^−^ no longer promotes additional complex formation.^27^ Based on this behaviour, a concentration of 4.0 mmol L^−1^ is recommended as the optimal condition for future studies involving continuous absorbance monitoring of the FeSCN^2+^ complex. The trend is illustrated in Fig. 4, showing the absorbance profile across the SCN^−^ concentration range.

#### Effect of hydrochloric acid concentration

**Fig. 5:**
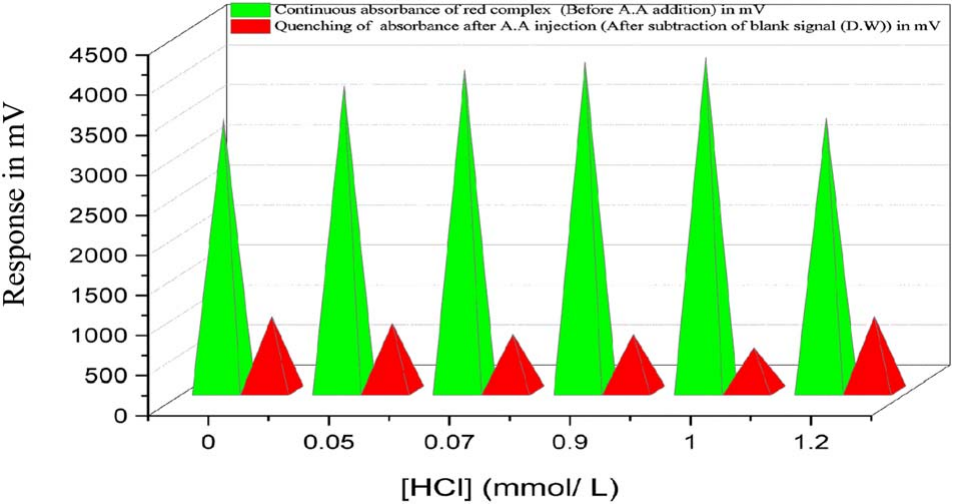
Effect of hydrochloric acid concentration on absorbance of complex formation and optimal concentration of HCl = 1.0 mmol L^−1^.

A fixed concentration of Fe^3+^ (2.0 mmol L^−1^) was prepared in varying concentrations of hydrochloric acid (HCl) ranging from 0.05 to 1.2 mmol L^−1^ and passed through the first channel of the flow injection system as a blank solution delivered through the carrier stream instead of distilled water. Simultaneously, a constant and optimal concentration of SCN^−^ (4.0 mmol L^−1^) was introduced through the second channel. The continuous absorbance of the FeSCN^2+^ complex increased with rising HCl concentration, reaching a maximum at 1.0 mmol L^−1^. Beyond this concentration, a decline in absorbance was observed. This behaviour may be attributed to the increased protonation of SCN^−^ at higher acid concentrations, which reduces its availability for complexation with Fe^3+^. Additionally, increased ionic strength and competitive chloride coordination may interfere with the formation of FeSCN^2+^. Fig. 5 presents the variation in FeSCN^2+^ complex absorbance as a function of HCl concentration.

#### Effect of sample volume and flow rate on ascorbic acid quenching efficiency

**Fig. 6:**
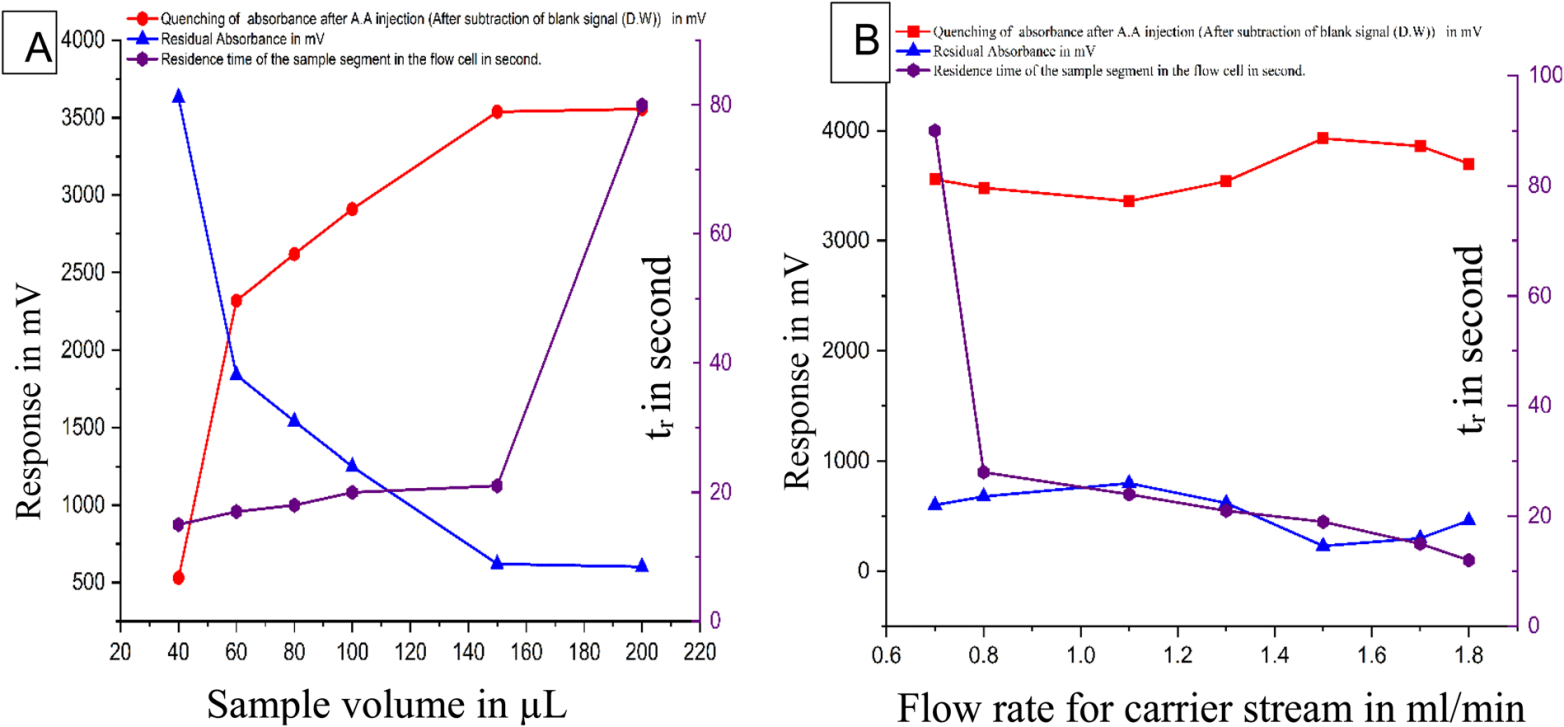
Effect of (A) sample volume and (B) flow rate on the quenching efficiency of the red FeSCN^2+^ complex by ascorbic acid using Fe^3+^ (2 mmol L^−1^)–SCN^−^ (4 mmol L^−1^)–H_3_O^+^ (1 mmol L^−1^) system in the prescence of [A.A] = 38 mmol L^−1^.

This study uses a flow injection system to investigate how the volume of the sample segment influences ascorbic acid's ability to quench the continuous absorbance of the red-coloured FeSCN^2+^ complex. Volumes ranging from 40 to 200 μL were tested, revealing a progressive increase in quenching efficiency with larger sample segments, reaching a maximum effect at 150 μL. Beyond this volume, no further enhancement was observed; instead, broader signal profiles emerged at both the apex and base width. It is likely attributed to the increased cross-sectional exposure of the ascorbic acid segment to the detection path, coupled with its prolonged residence time within the measurement cell. These factors collectively facilitate a more extensive interaction with the coloured species, enhancing the quenching of continuous absorbance up to a specific threshold. Further increases in sample volume beyond this point do not yield additional benefit and instead result in signal broadening. Based on these observations, 150 μL was identified as the optimal volume, providing effective quenching while maintaining signal clarity and measurement repeatability (Fig. 6A). And to balance reagent economy and improve analytical sensitivity, the effect of flow rate on the ability of ascorbic acid to quench the continuous absorbance of the red-coloured FeSCN^2+^ complex was studied. The flow rate was adjusted by varying the speed of the peristaltic pump from 5 to 35 rpm, corresponding to 0.7–1.8 mL min^−1^ for the carrier stream and 0.9–2.0 mL min^−1^ for both reagent lines: ferric ion and thiocyanate ion. The results indicated that higher flow rates led to a decrease in the quenching ability of ascorbic acid. This reduction is likely due to stronger physical effects at higher speeds, especially dilution and dispersion caused by convective flow. These effects reduce the contact time and concentration of the quencher in the detection area, limiting its ability to suppress the absorption of the coloured complex. The best quenching performance was observed at flow rates of 1.6 mL min^−1^ for both reagent lines and 1.5 mL min^−1^ for the carrier stream (Fig. 6B). These conditions provided a satisfactory balance between chemical efficiency and stable signal response while minimising reagent consumption.

#### Evaluation of mixing coil designs for optimal complex formation and quenching efficiency in flow injection systems

**Fig. 7:**
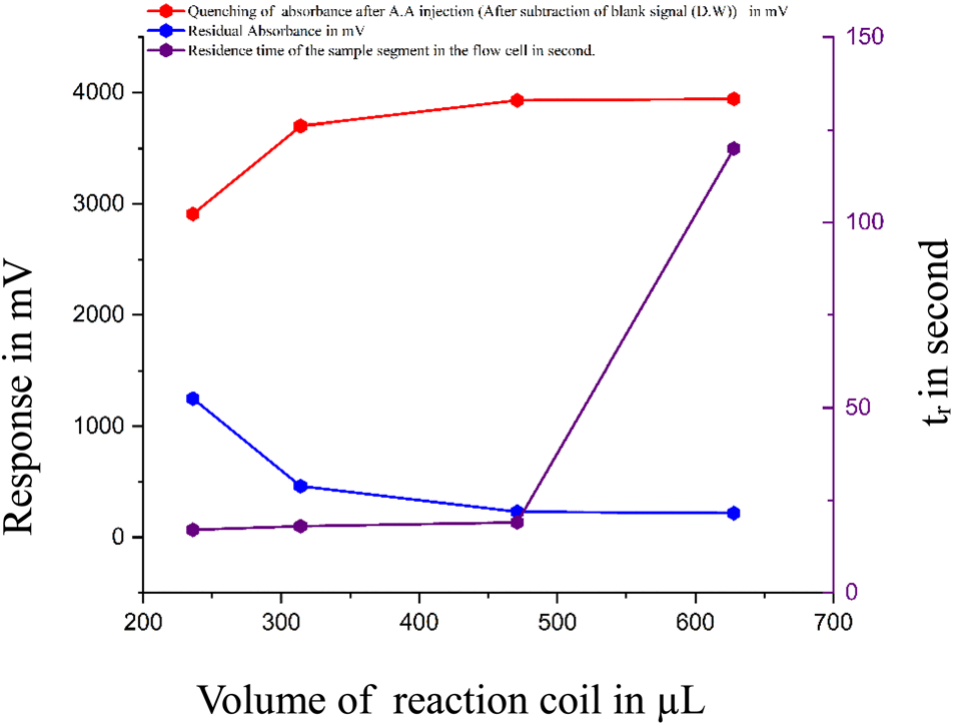
Effect of volume of reaction coil on the absorbance of complex formation, quenching by ascorbic acid, residual absorbance and the optimum volume was determined to be 471 μL.

The mixing coil is essential for forming the red FeSCN^2+^ complex in an acidic medium supplied by hydrochloric acid. In this study, the ferric ion stream in the acid was first combined with thiocyanate ions and then passed through a mixing coil to complete the reaction and produce a uniform-colored segment, which was later merged with ascorbic acid to initiate quenching. After optimising parameters such as flow rate, sample volume, and reagent concentration, various locally fabricated coils (lengths of 30–80 cm; made of PTFE and silicone) were tested. PTFE offered better chemical stability and mixing, while silicone provided flexibility with slightly lower efficiency. Longer coils caused signal broadening and reduced sensitivity due to sample dispersion. A 60 cm (471 μL) PTFE coil gave the best results (Fig. 7), enabling full complex formation and a sharp quenching response. These results underscore the critical role of coil design in optimising reaction kinetics and ensuring accuracy in flow injection systems.

#### Evaluation of linearity, precision, and detection capability

**Fig. 8:**
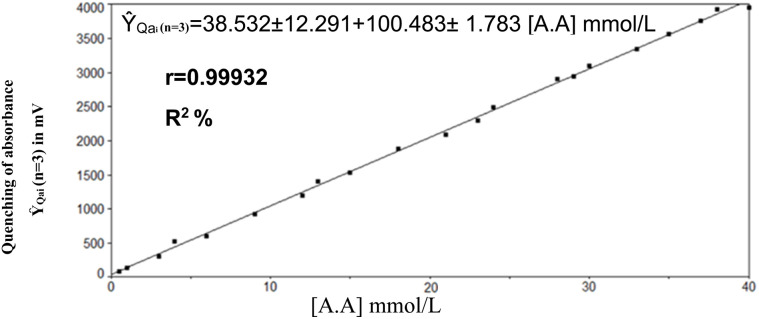
The effect of varying ascorbic acid concentration on enhancing the quenching of the red complex's absorbance, as visualized through a calibration curve constructed.

Standard ascorbic acid solutions varying from 0.5 to 45 mmol L^−1^ were prepared and injected with a fixed volume of 150 μL, while all chemical and physical parameters were maintained constant (Fe^3+^ at 2 mmol L^−1^, –SCN^−^ at 4 mmol L^−1^, and –H_3_O^+^ at 1 mmol L^−1^, with flow rates of 1.6 mL min^−1^ for both reagent lines and 1.5 mL min^−1^ for the carrier stream, and a sample volume of 150 μL). As the concentration of ascorbic acid increased, the absorbance of the red FeSCN^2+^ complex diminished progressively, indicating successful quenching. A calibration curve was constructed up to 40 mmol L^−1^ with a correlation coefficient of *r*^2^ = 0.9986 (Fig. 8), derived from 15 independently prepared and measured standard concentrations (*n* = 15). The linear regression equation is *Ŷ*_Qai_ (*n* = 3) (mV) = 38.532 ± 12.291 + 100.483 ± 1.783 [A.A] mmol L^−1^. The approach attained a detection limit of 0.05 mmol L^−1^ (1.3209 μg per sample) and a limit of quantification (LOQ) of 131.0791 μg per sample. Above 40 mmol L^−1^, linearity started to decline, perhaps due to saturation of the reaction site or other chemical interactions affecting the quenching process. The data indicated that the concentration responsible for approximately reducing the complex's absorbance is 40 mmol L^−1^. Intra-day repeatability was employed to evaluate technique precision, utilising eight replicated injections at concentrations of 12 and 30 mmol L^−1^. The relative standard deviation (RSD) was below 1%, signifying excellent short-term precision. Inter-day repeatability was assessed by assessing identical concentrations over various days, yielding RSD values below 2%, signifying significant procedure reliability.

#### Interference effect

To evaluate the analytical specificity of the proposed CFIA method, interference testing was conducted at a fixed ascorbic acid concentration of 10 mmol L^−1^ in the presence of commonly encountered excipients and ions, including glucose, citric acid, sucrose, starch, magnesium stearate, talc, sodium benzoate, sorbitol, lactose. Each interferent was added at a concentration of 2 mmol L^−1^ to simulate real formulation conditions. Each compound was tested individually at concentrations commonly found in pharmaceutical preparations. The percentage interference was calculated by comparing the absorbance change relative to the blank and standard ascorbic acid signal. The results summarized in Table 1 indicated that none of these substances caused significant interference. The calculated interference percentages were consistently close to zero, indicating excellent selectivity of the proposed method toward ascorbic acid.

**Table 1:** Interference study results table^a^

Interfering substance	Typical use in formulations	Tested concentration	% interference
Glucose	Sweetener/filler	1.0 mmol L^−1^	∼0.2%
Citric acid	pH adjuster/preservative	0.5 mmol L^−1^	∼0.3%
Sucrose	Sweetener	1.0 mmol L^−1^	∼0.1%
Starch	Binder/filler	0.2% w/v	∼0.0%
Magnesium stearate	Lubricant	0.1% w/v	∼0.0%
Talc	Anti-caking agent	0.1% w/v	∼0.0%
Sodium benzoate	Preservative	0.5 mmol L^−1^	∼0.2%
Sorbitol	Sweetener/stabilizer	1.0 mmol L^−1^	∼0.1%
Lactose	Filler/stabilizer	1.0 mmol L^−1^	∼0.2%

a All interference percentages were calculated relative to the standard absorbance signal of FeSCN^2+^ complex. No significant interference was observed; values remained consistently near zero.

#### Evaluation of analytical performance of flow injection versus conventional UV and HPLC techniques for ascorbic acid quantification

For the quantitative measurement of ascorbic acid, the suggested quenching-based flow injection approach was thoroughly contrasted with two well-established analytical techniques, high-performance liquid chromatography (HPLC) and UV spectrophotometry. The spectrophotometric analysis performed at *λ* = 265 nm demonstrated a linear concentration range of 1–10 mmol L^−1^, with a limit of detection (LOD) at 0.5 mmol L^−1^, suggesting a limit of detection and a narrower working range.^28^ The HPLC method employed a Kinetex C18 reversed-phase column (100 mm × 4.6 mm, 2.6 μm particle size) under isocratic conditions. The mobile phase consisted of a 0.05 M potassium dihydrogen phosphate buffer (pH 3.5), acetonitrile, and methanol in a 70 : 20 : 10 v/v/v ratio. The stationary phase (C18) assisted hydrophobic interactions, whereas the mobile phase contributed to the effective elution and resolution of ascorbic acid. Detection was conducted at 255 nm utilising a UV detector. This approach exhibited a linear range of 0.5–20 mmol L^−1^, a LOD of 0.2 mmol L^−1^, with a flow rate of 1.0 mL min^−1^ and an injection volume of 10 μL, providing enhanced sensitivity and an expanded quantification range.^29,30^ The findings highlight the exceptional analytical capabilities of the developed flow injection method, showcasing its sensitivity, reliability, and linear dynamic range. This positions it as a strong alternative for the swift and automated measurement of ascorbic acid across different sample matrices.^31^

#### Comparative application of flow injection, UV spectrophotometric, and HPLC methods for the determination of ascorbic acid in commercial pharmaceutical samples

To evaluate the applicability of the developed quenching-based flow injection method, three pharmaceutical formulations containing 500 mg of ascorbic acid were selected from different Iraqi manufacturers: Furat Pharma, Kindi Pharma, and Aljazeera. From each product, 4 mL was transferred into a 10 mL volumetric flask and spiked with incremental volumes (0.0, 0.4, 0.6, 0.8, and 1.0 mL) of a 50 mmol per L standard ascorbic acid solution, yielding final concentrations of 0, 2, 3.0, 4.0, and 5.0 mmol L^−1^. The unspiked flask served as the sample matrix. Quantification was performed using the standard addition method, and results were obtained using the developed flow injection technique, UV spectrophotometry, and HPLC. From Table 2, it can be concluded that all analytical methods employed—namely Flow Injection Analysis (FIA), UV-spectrophotometry, and high-performance.

**Table 2:** Analytical evaluation of commercial ascorbic acid samples with reliability indicators^a^

Commercial pharmaceutical formulations (Iraq)	Nominal content (mg)	Flow injection (mg ± CI)	UV (mg ± SD)	HPLC (mg ± SD)	Recovery (%) flow/UV/HPLC	*T*-test result	*F*-test (ANOVA)
Furat Pharma	500.0	516.745 ± 7.382	518.0 ± 7.565	517.088 ± 6.890	103.35/103.6/103.42	Flow & UV *t*_cal_ (1.205) < *t*_tab_ (4.303)	*F* < *F*_a_ 0.0136 < 5.14
Kindi Pharma	500.0	496.188 ± 1.242	491.5 ± 1.532	495.5 ± 1.612	99.24/98.3/99.1		
						Flow & HPLC *t*_cal_ (1.149) < *t*_tab_ (4.303)	
Aljazeera Pharma	500.0	488.613 ± 5.212	485.671 ± 5.675	486.435 ± 4.675	97.72/97.13/97.29		

a *δ*_*n*−1_: standard deviation based on replicate measurements (*n* = 3). *T*-test and ANOVA performed at *α* = 0.05; critical values from standard statistical tables. 
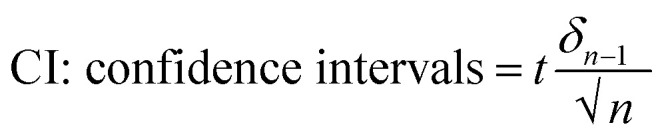
 calculated at 95% confidence level based on replicate measurements (*n* = 3). Statistical tests performed at *α* = 0.05.

From Table 1, it can be concluded that all analytical methods employed—namely Flow Injection Analysis (FIA), UV-spectrophotometr,^32^ and High-Performance Liquid Chromatography (HPLC)^33^—provided results closely aligned with the nominal ascorbic acid content of 500 mg. The measured concentrations ranged from approximately 485.7 to 518.0 mg, with recovery percentages spanning 97.13% to 103.6%, indicating satisfactory accuracy and compliance with pharmacopeial standards. The calculated confidence intervals and low standard deviations from triplicate measurements demonstrate high precision and reproducibility. Moreover, statistical analysis using paired *t*-tests and one-way ANOVA confirmed no significant differences between the methods (*t*_cal_ < *t*_tab_; *F*_cal_ < *F*_tab_ at *α* = 0.05), supporting the consistency and equivalency of the techniques. These findings collectively validate the reliability of FIA, UV, and HPLC as suitable and interchangeable approaches for routine quantification of ascorbic acid in commercial pharmaceutical formulations under quality control conditions.

To further demonstrate the versatility and broader applicability of the developed CFIA method, the protocol was extended to the quantification of ascorbic acid in food matrices, specifically three commercially available orange juice products: Sunquick Orange Concentrate, Rani Orange Juice, and Almarai Natural Orange Juice (Table 3). Each sample underwent standardized pre-treatment involving 1 : 10 dilution with distilled water, filtration using Whatman filter paper to remove suspended solids, and storage at room temperature away from light to prevent oxidative degradation of vitamin C. Analytical measurements were performed using the optimized CFIA system, and results were compared with those obtained *via* conventional UV-spectrophotometry and HPLC to assess method consistency across different matrices.

**Table 3:** Ascorbic acid quantification in orange juice samples

Sample name	Vitamin C (mg L^−1^) CFIA	Vitamin C (mg L^−1^) UV-Vis	Vitamin C (mg L^−1^) HPLC	Recovery (%) CFIA
Sunquick orange	420.5	415.2	418.7	101.2
Rani orange	312.8	308.6	310.4	100.8
Almarai natural orange	256.3	250.7	254.9	100.6

As shown in Table 3, the CFIA method yielded ascorbic acid concentrations that were in close agreement with those obtained *via* UV and HPLC, with recovery percentages ranging from 100.6% to 101.2%. These values reflect excellent analytical accuracy and minimal matrix interference, even within complex food systems. The consistency across methods highlights the robustness of the CFIA approach, which demonstrated high sensitivity, precision, and operational simplicity under ambient conditions. These results confirm that the CFIA method is not only suitable for pharmaceutical applications but also adaptable to food matrices, positioning it as a promising candidate for routine quality control of ascorbic acid in both industrial and nutritional contexts.

The potential impact of inherent sample coloration on spectrophotometric measurements is a valid concern, particularly when analyzing food and environmental matrices that may contain chromophoric species contributing to background absorbance or spectral interference. To address this, the optical detection system integrated into the developed CFIA protocol was meticulously engineered to mitigate such effects and enhance analytical selectivity.

The system utilizes eight blue light-emitting diodes (LEDs) arranged in a matrix geometry distributed across two angular regions (0–90° and 0–180°), with dual solar cells serving as photonic energy transducers. Spectral acquisition was optimized at the 0–180° angular configuration, which demonstrated superior absorbance stability and signal-to-noise ratio compared to conventional linear arrangements. The use of blue LEDs, emitting within a narrow spectral range (470–490 nm), ensures targeted excitation of the [Fe(SCN)]^2+^ complex while minimizing susceptibility to absorbance contributions from other colored species that typically absorb in broader or different regions of the spectrum.

Additionally, the dual solar cell configuration enables differential detection, allowing the system to distinguish analyte-specific absorbance changes from matrix-induced background coloration. This design feature significantly reduces the influence of sample color on analytical accuracy.

Empirical validation was conducted using colored juice samples, including orange-based matrices, which inherently possess strong pigmentation. The CFIA system maintained consistent baseline absorbance and yielded recovery values within acceptable limits, demonstrating its robustness in the presence of moderate coloration. These results affirm the system's capability to perform reliably in real-world conditions without requiring extensive sample decolorization or background correction.

Nonetheless, we acknowledge that extreme coloration or turbidity may still pose analytical challenges. Future work will explore the integration of multi-wavelength referencing and algorithmic baseline correction to further enhance the system's resilience against complex matrix effects, thereby extending its applicability to a broader range of sample types.

## Discussion

The developed CFIA system demonstrated excellent analytical efficiency, enabling the processing of up to 55 samples per hour using micro-volumes of 150 μL, which contributes to a substantial reduction in chemical reagent consumption, analysis time, and overall operational cost. The method exhibited a wide linear range and low detection limits reaching the microgram level, outperforming conventional spectrophotometric and chromatographic techniques in sensitivity and speed. Measurements were conducted using a locally designed spectrophotometric unit that incorporates eight blue LED sources positioned at 0–90° and eight at 0–180°, enabling multidirectional angular irradiation. Optical signals were detected by a dual of solar cells, and measurements optimised at 0–180° yielded sharp, noise-free responses that enhanced both sensitivity and reproducibility. The modular structure and precision of this optical system allow for flexible adaptation to a wide range of chromogenic and redox-active analytes beyond ascorbic acid. To illustrate the advantages of the proposed method, Table 4 provides a comparison against three conventional techniques, and summarises key analytical parameters. The CFIA protocol offers superior detection performance, economic operation, and broader applicability in pharmaceutical analysis, environmental, and food matrices.

**Table 4:** Comparative overview of analytical methods for ascorbic acid determination

Method	Reagents used	Calibration range	Detection limit	Reference
UV spectrophotometry	Methanol : water (50 : 50), *λ* = 258 nm	3–15 μg mL^−1^	0.5 μg mL^−1^	34
Catalytic titration	Cr(vi), Mn(ii), acidic medium	0.001–0.25 mg mL^−1^	0.00154 mg mL^−1^	20
FTIR spectroscopy	KBr matrix, solid-state scan	2–100 μg mL^−1^	0.3 μg mL^−1^	35
Spectrophotometric (malate)	Malate buffer, metaphosphoric acid	0–6.95 mM	0.236 mM	28
Proposed CFIA method	Fe^3+^, SCN^−^, H_3_O^+^, LED-based detection	0.5–40 mmol L^−1^	0.05 mmol L^−1^ (1.32 μg)	Present study

It is important to note that the developed CFIA protocol is classified as a destructive analytical technique, as it involves an irreversible redox reaction between the analyte (ascorbic acid) and the chromogenic reagent system composed of ferric (Fe^3+^) and thiocyanate (SCN^−^) ions. Upon injection into the flow stream, ascorbic acid reduces the *in situ* formed [Fe(SCN)]^2+^ complex, leading to a measurable decrease in absorbance intensity. This chemical transformation consumes the analyte during detection, and the sample cannot be recovered post-analysis. The continuous flow design further reinforces this classification, as each aliquot is mixed with reagents and directed toward the detection unit, resulting in permanent alteration of the sample. This clarification has been incorporated to ensure transparency and completeness in describing the operational characteristics and limitations of the method.

The proposed method demonstrated superior sensitivity, achieving a detection limit of 0.05 mmol L^−1^, which is significantly lower than that of conventional spectrophotometry (0.5 mmol L^−1^) and HPLC (0.2 mmol L^−1^). This enhanced performance is attributed to the unique design of the homemade photometric system, which incorporates a tubular flow cell (2 mm internal diameter, 100 mm length) housed within a brass incubator. The system features dual-angle irradiation (0–90°) and detection (0–180°) sites, allowing flexible optical alignment and minimizing spectral noise through self-absorption and symmetry interference control.

The continuous flow setup ensures a stable baseline and sharp signal quenching upon sample injection, contributing to high repeatability and resolution. These structural and operational advantages collectively enable the method to detect lower concentrations of ascorbic acid with high reliability. All results were experimentally obtained by the authors, and the cited references serve only to support the theoretical framework of the employed techniques.

In addition, The effect of temperature was not investigated in this study due to the inherent nature of the chemical reaction employed. The formation of the red-colored complex [Fe(SCN)]^2+^ from the reaction between ferric ion and thiocyanate ion occurs rapidly at ambient temperature without the need for external heating. Experimental observations confirmed that room temperature is sufficient to ensure complex formation and stable absorbance.

Furthermore, the determination of ascorbic acid was based on its quenching effect, which relies on its reducing capability to convert ferric ion to ferrous ion, thereby decreasing the absorbance of the complex. Ascorbic acid is known to be thermally sensitive, and elevated temperatures may adversely affect its quenching efficiency, potentially compromising the accuracy of the results.

Therefore, conducting the analysis at room temperature provides optimal conditions for the reaction, ensures reproducibility of the method, and preserves the integrity of the analyte by avoiding any thermal degradation.

## Conclusions

A novel CFIA-based spectrophotometric method was successfully developed and validated for the quantification of ascorbic acid utilising a homemade optical detecting equipment. The system exhibited exceptional sensitivity (LOD = 0.05 mmol L^−1^), extensive linearity (0.5–40 mmol L^−1^), and superior precision (RSD < 2%), surpassing traditional UV and HPLC techniques in speed, reagent efficiency, and analytical throughput. The redox-induced quenching technique utilising the FeSCN^2+^ complex facilitated selective and interference-free detection. The application of the approach to commercial pharmaceutical goods validated its reliability and robustness, positioning it as a viable alternative for the routine quality control of vitamin C formulations.

## Author contributions

Turkey N. S. conceptualization, data curation, formal analysis, funding acquisition, investigation methodology project administration, supervision, writing – original draft, resources. Ghufran K. Allawi software, validation visualization, writing – review & editing.

## Conflicts of interest

There are no conflicts to declare.

## Supplementary Material

RA-015-D5RA07160G-s001

## Data Availability

The data supporting this article have been included as part of the supplementary information (SI). Supplementary information is available. See DOI: https://doi.org/10.1039/d5ra07160g.
