# Supplementary material for: Development of a multi-source blue light irradiation platform for photometric quantification of ascorbic acid via continuous absorbance quenching of the ferric–thiocyanate complex
Source: RSC Adv. 2025 Oct 24;15(48):40655–65. doi: 10.1039/d5ra07160g (PMC12550886; doi:10.1039/d5ra07160g)
Supplement: RA-015-D5RA07160G-s001 [file RA-015-D5RA07160G-s001.pdf]

This journal is © The Royal Society of Chemistry 2025

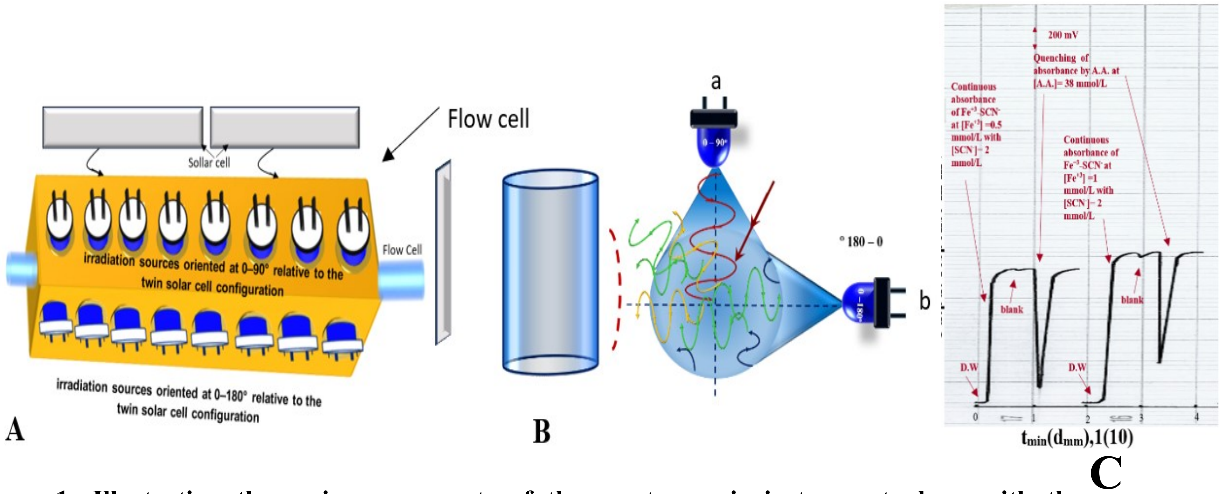

Figure 1: Illustrating the main components of the spectroscopic instrument along with the measurement configuration: A- measurement cell, B- Illuminated area by: a) Source at 0–90°, b) Source at 0–180 , C: real profile measured responses obtained from the chart recorder (T : Tim in minute, d: distance in ml meter).

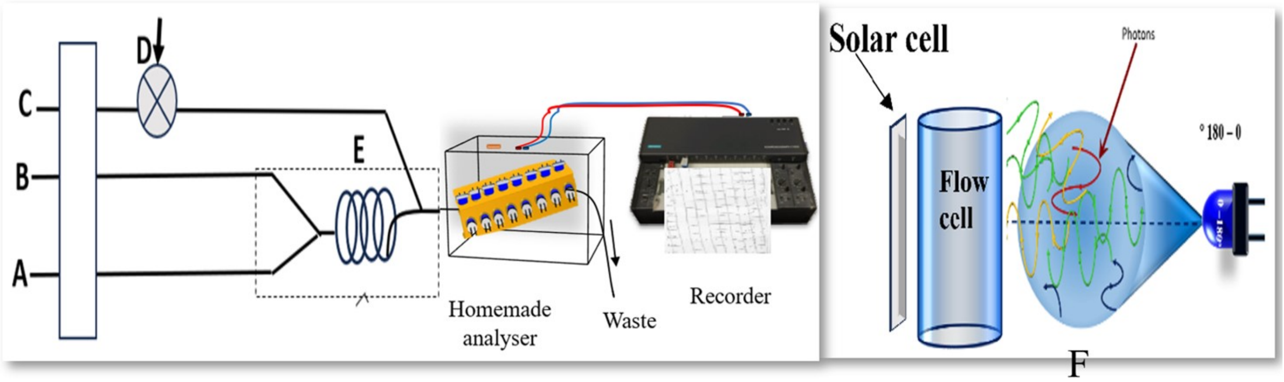

Figure 2: Diagram of the continuous flow injection system. A: Line moving ferric ion in hydrochloric acid medium; B: Line carrying thiocyanate ion; C: Carrier stream containing ascorbic acid; D: Injection valve for sample introduction; E: The unit mixes reagents to create the red $FeSCN^{2+}$ complex. This stage is followed by a reaction coil for complex development, which intersects line C to quench the complex with ascorbic acid prior to photometric detection .F: The LED-blue irradiation source is shown with the single solar cell detector, which is positioned at a measuring angle of 0° to 180°.

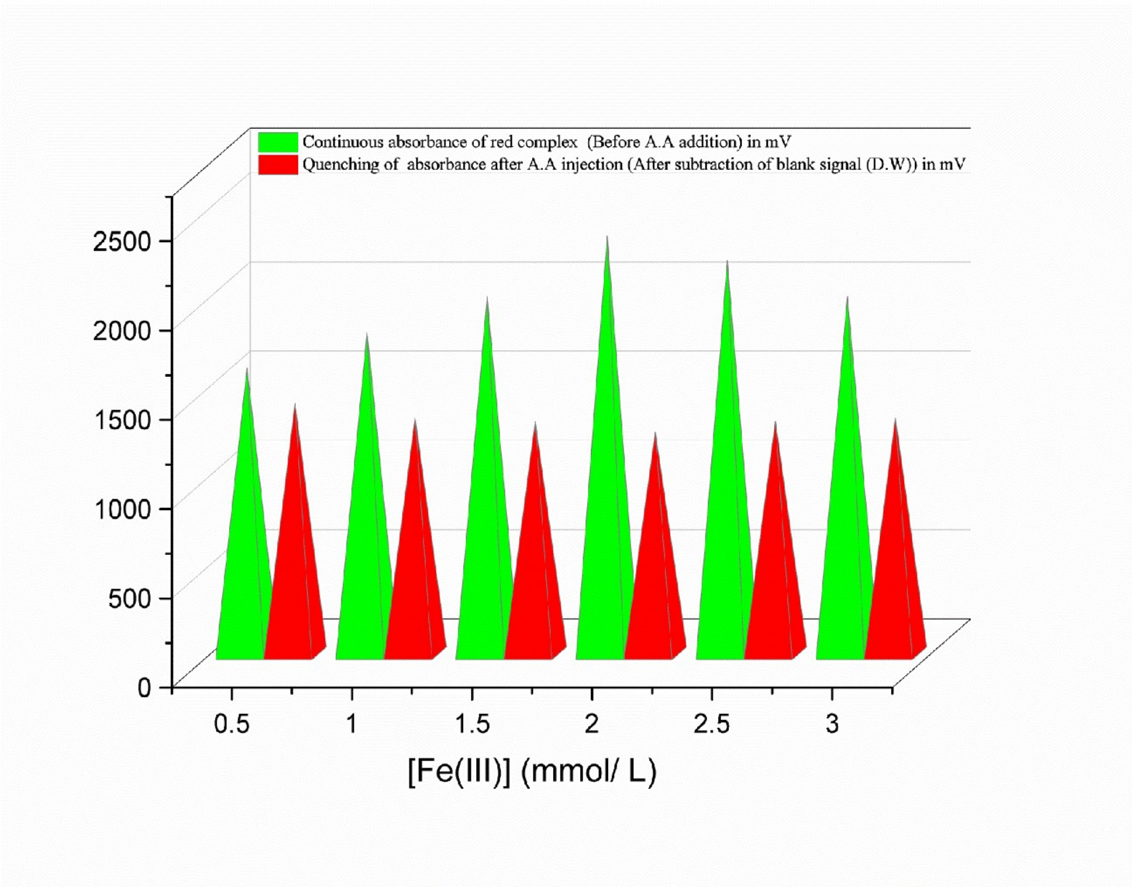

Figure 3: Effect of Ferric Ion Concentration on absorbance of Complex Formation , Quenching Sensitivity by Ascorbic Acid, Residual Absorbance and the optimal concentration of ferric ion = 2.0 mmol/L

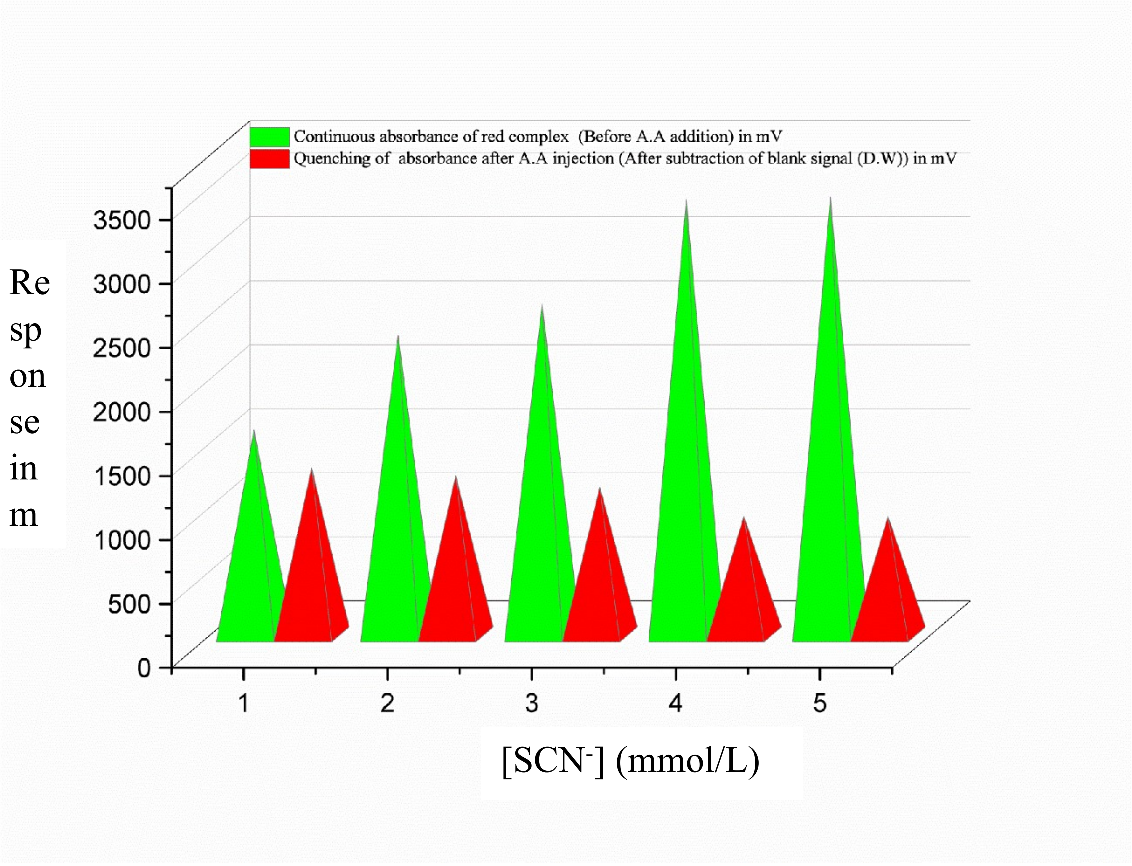

Figure 4: Effect of Thiocyanate Ion Concentration using Fe^{+3} (2 mmol/L)-SCN-(variable concentration)-H_2O system and [A.A]= 38 mmol/L on absorbance of Complex Formation, Quenching by Ascorbic Acid , Residual Absorbance and the optimal concentration of SCN^{-}= 4.0 mmol/L

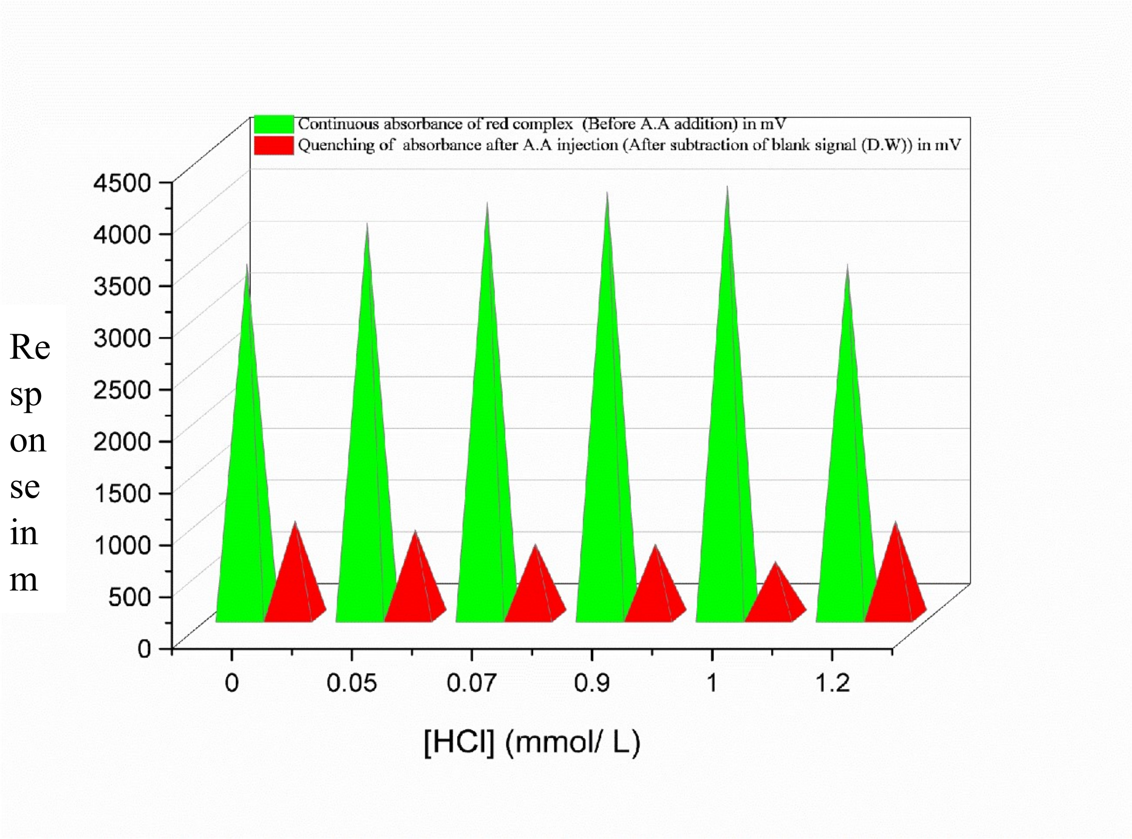

Figure 5: Effect of Hydrochloric Acid Concentration on absorbance of Complex Formation and the Residual Absorbance, optimal concentration of HCl= 1.0 mmol/L

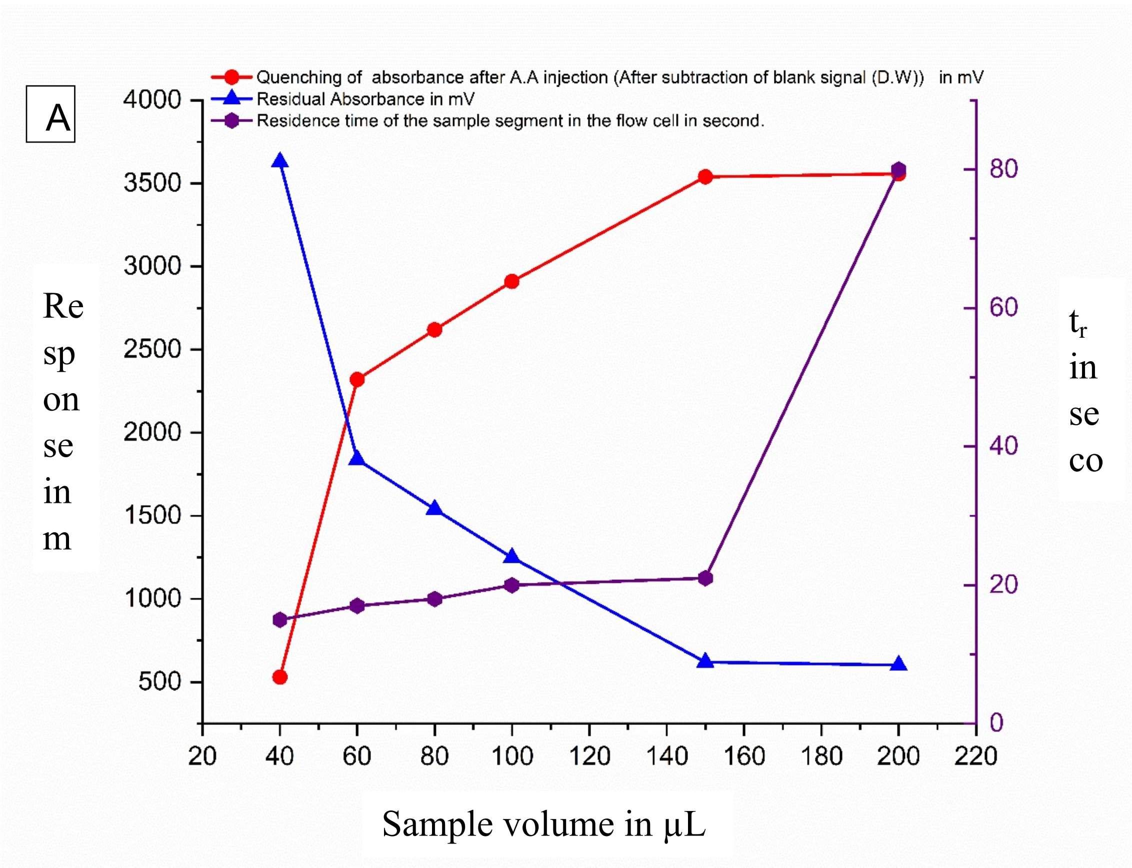

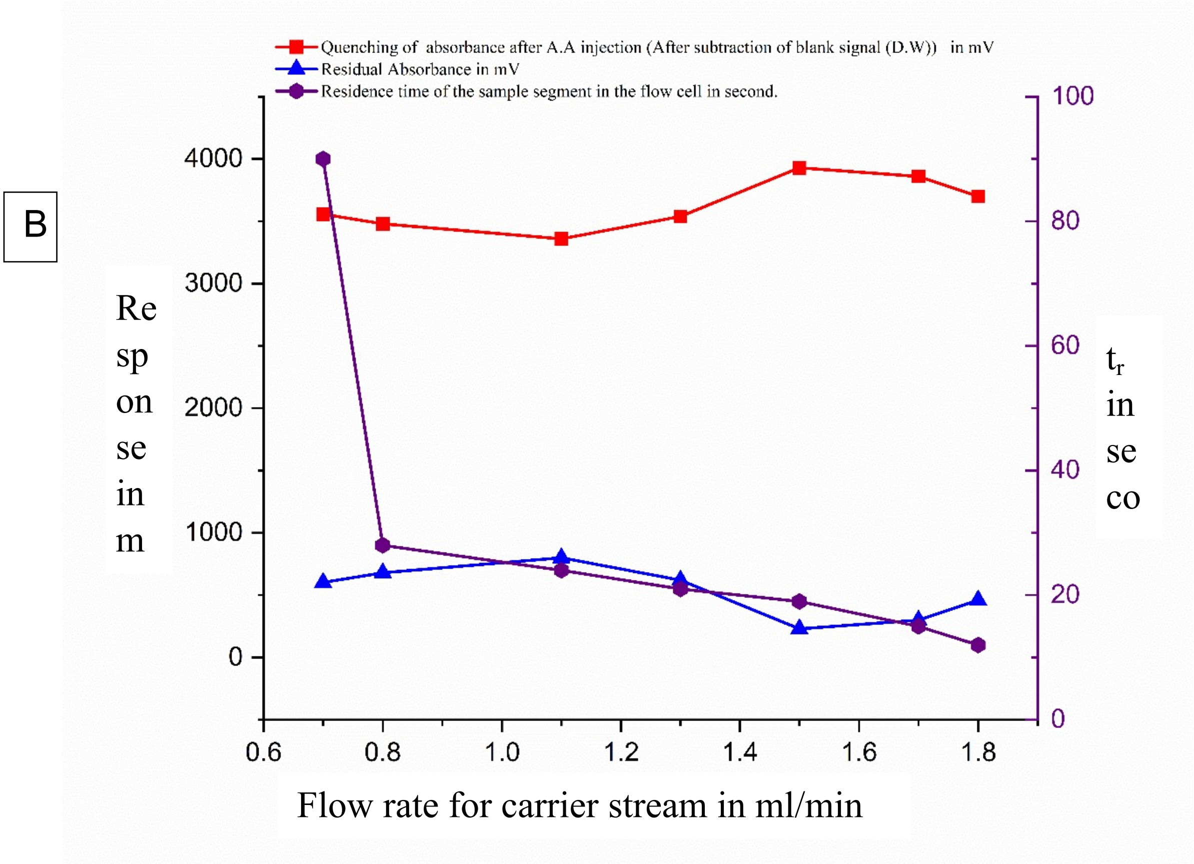

Figure 6: Effect of A- Sample Volume and B- Flow Rate on the Quenching Efficiency of the Red $FeSCN^{2+}$ Complex by Ascorbic Acid using Fe^{+3}(2 mmol/L) -SCN^{-}(4 mmol/L) - H_3O^{+} (1 mmol/L) system in the Prescence of [A.A]= 38 mmol/L

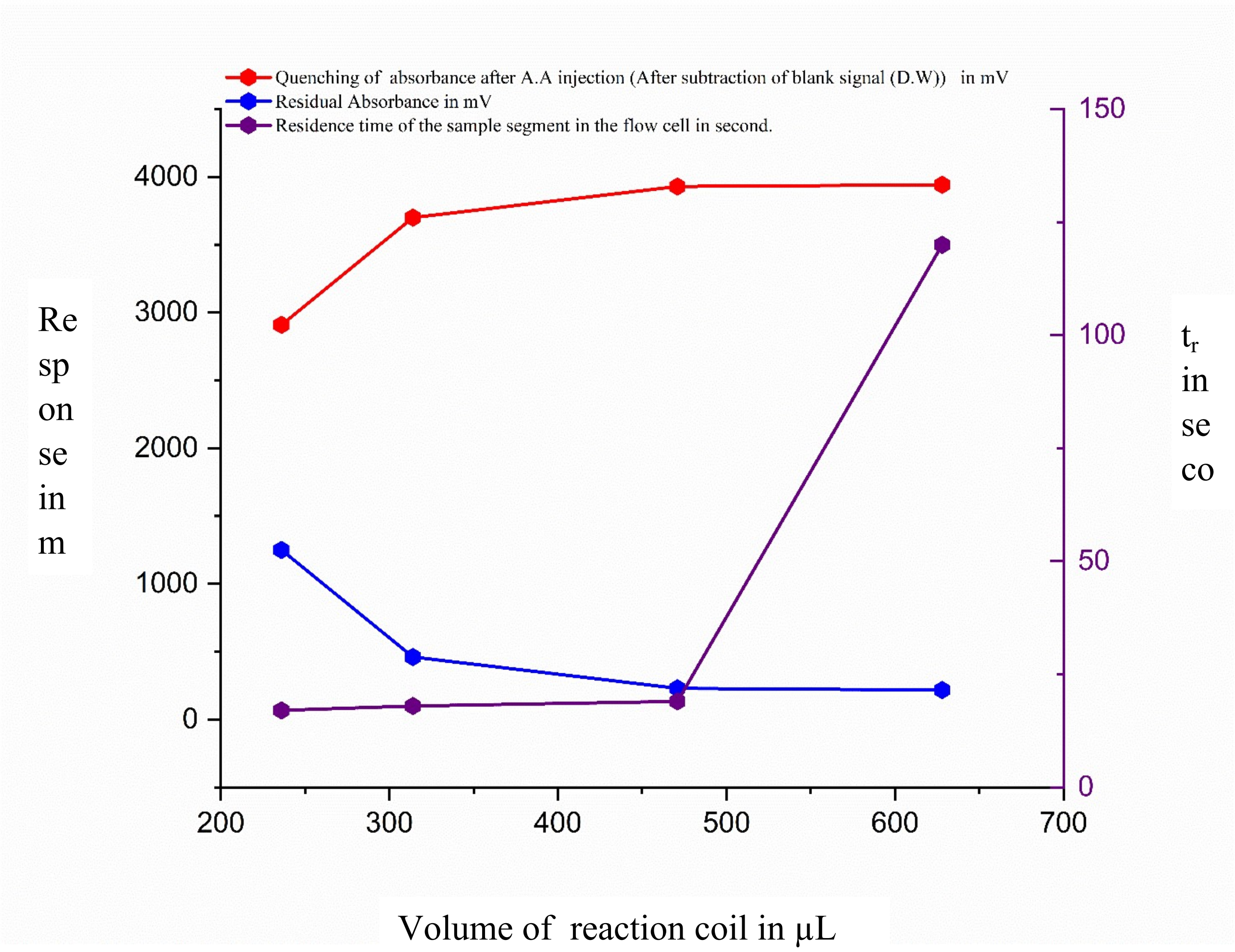

Figure 7: B- Effect of Volume of reaction coil on the absorbance of Complex Formation, Quenching by Ascorbic Acid , Residual Absorbance and the optimum volume was determined to be 471 µL

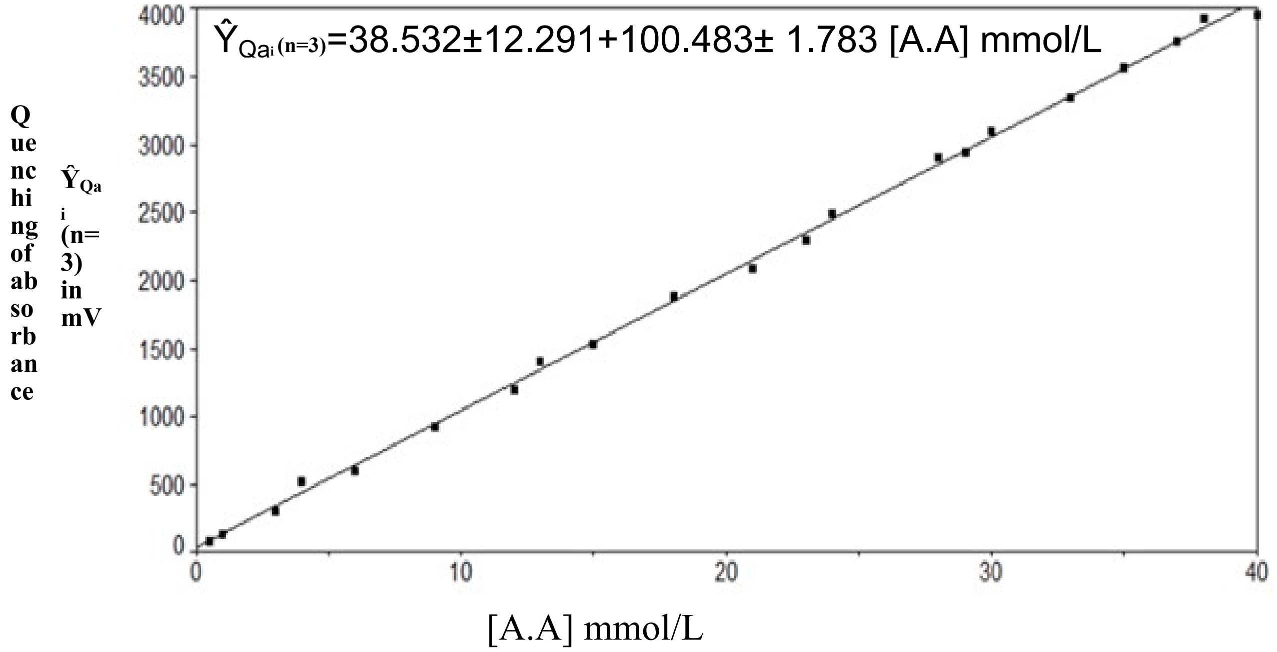

Figure 8 :The effect of varying ascorbic acid concentration on enhancing the quenching of the red complex's absorbance, as visualized through a calibration curve constructed.
